# Increased Cleavage of Japanese Encephalitis Virus prM Protein Promotes Viral Replication but Attenuates Virulence

**DOI:** 10.1128/spectrum.01417-22

**Published:** 2022-06-13

**Authors:** Junyao Xiong, Mengxue Yan, Shuo Zhu, Bohan Zheng, Ning Wei, Lingen Yang, Youhui Si, Shengbo Cao, Jing Ye

**Affiliations:** a State Key Laboratory of Agricultural Microbiology, Huazhong Agricultural University, Wuhan, Hubei, China; b Laboratory of Animal Virology, College of Veterinary Medicine, Huazhong Agricultural University, Wuhan, Hubei, People's Republic of China; c The Cooperative Innovation Center for Sustainable Pig Production, Huazhong Agricultural University, Wuhan, Hubei, People's Republic of China; Changchun Veterinary Research Institute

**Keywords:** JEV, prM, furin, replication, virulence

## Abstract

In flavivirus, the furin-mediated cleavage of prM is mandatory to produce infectious particles, and the immature particles containing uncleaved prM cannot undergo membrane fusion and release to the extracellular environment. However, the detailed relationship between viral replication or pathogenicity and furin in Japanese encephalitis virus (JEV) hasn’t been clarified. Here, JEV with the mutations in furin cleavage sites and its nearby were constructed. Compared with WT virus, the mutant virus showed enhanced cleavage efficiency of prM protein and increased replication ability. Furthermore, we found that the mutations mainly promote genomic replication and assembly of JEV. However, the mutant formed smaller plaques than WT virus in plaque forming assay, indicating the lower cytopathogenicity of mutant virus. To assess the virulence of JEV mutant, an *in vivo* assay was performed using a mouse model. A higher survival rate and attenuated neuroinflammation were observed in JEV mutant-infected mice than those of WT-infected mice, suggesting the cleavage of prM by furin was closely related to viral virulence. These findings will provide new understanding on JEV pathogenesis and contribute to the development of novel JEV vaccines.

**IMPORTANCE** Japanese encephalitis virus (JEV) is the leading cause of viral encephalitis epidemics in Southeast Asia, affecting mostly children, with high morbidity and mortality. During the viral maturation process, prM is cleaved into M by the cellular endoprotease furin in the acidic secretory system. After cleavage of the prM protein, mature virions are exocytosed. Here, the mutant in furin cleavage sites and its nearby was constructed, and the results showed that the mutant virus with enhanced replication mainly occurred in the process of genomic replication and assembly. Meanwhile, the mutant showed an attenuated virulence than WT virus *in vivo*. Our study contributes to understanding the function of prM and M proteins and provides new clues for live vaccine designation for JEV.

## INTRODUCTION

Japanese encephalitis (JE), caused by Japanese encephalitis virus (JEV), is one of the most important viral encephalitis in East and Southeast Asia (1–3). Annually, more than 68,000 JE cases were reported where fatality rate can reach up to 30%, and the 20% to 30% of survivors can suffer permanent neurologic sequelae, such as inability to speak, recurrent seizures, or paralysis (4–6). JEV is an enveloped, single-stranded and positive-sense RNA virus. The JEV genome encodes a single polyprotein that is hydrolyzed by cellular and viral proteases into three structural proteins (core [C], premembrane [prM], membrane [M], and envelope [E]) and seven nonstructural (NS) proteins (NS1, NS2A, NS2B, NS3, NS4A, NS4B, and NS5) (7, 8). NS proteins are mainly involved in viral replication and evasion from host immune response, while structural proteins are responsible for virus assembly and viral entrance into and exit from host cells.

During the life cycle in flavivirus, the immature flavivirus transits through the secretory pathway, and the decrease in pH induces a rearrangement in the conformation of the membrane proteins that exposes the prM protein furin cleavage sites (9, 10). Subsequently, the endoprotease furin cleaves prM to M protein in an acidic environment of the trans-Golgi apparatus after recognition of the sequence R-X-R/K-R in all flaviviruses, and the mature particles become infectious and release in the extracellular environment by exocytosis (9–12). The prM cleaved by furin is mandatory to produce infectious particles, and the immature particles containing uncleaved prM cannot undergo membrane fusion and release to the extracellular environment (13, 14). In flaviviruses, furin-mediated cleavage of the prM protein is usually incomplete. A mixture of immature, partially mature and mature extracellular particles can be detected, and the balanced breakdown will affect viral replication or virulence (15). For JEV or Zika virus (ZIKV), the decanoyl-Arg-Val-Lys-Arg-chloromethylketone (CMK), a specific furin inhibitor that can inhibit prM cleavage, resulting in a significant decrease in viral replication (16). In the case of Dengue virus (DENV) or West Nile virus (WNV) infection, the specific furin inhibitors capable of inhibiting prM cleavage can block the replication of DENV and WNV, which can reduce viral titers up to 10,000-fold (17). In addition, the DENV-1/2 chimeric virus with enhanced prM cleavage could be more efficient in controlling viremia during viral challenge in macaques immunized with the mutant chimeric virus (15). Furthermore, the efficient prM cleavage appeared in vero-furin cells leading to a decrease in the sensitivity of the virus to the neutralizing antibody (16). However, the inner relationship or mechanism of furin cleavage and viral replication or virulence in JEV has not been clarified.

The cleavage of prM to M protein is mediated by endoprotease furin, depending on the recognition of the furin cleavage site motif (R-X-R/K-R). However, this pattern does not explain all furin cleavage sites (18–20). The multibasic amino acid motif is not a sufficient requirement for the efficient furin proteolysis of the substrate protein, and the amino acids located at the P7, P6, P5, P3, and P1’ to P4’ oppositions are very important for modulating furin cleavage efficiency (21). Previous studies in our laboratory have demonstrated that mutations in furin cleavage sites and its nearby lead to alterations in ZIKV replication and virulence (22). Therefore, the similar mutations in prM of JEV were constructed to evaluate the relationship between prM cleavage and viral replication or virulence in our study. The results showed that the prM-S78R-K79R-S81R mutations promoted the cleavage of prM protein and increased viral replication by facilitating viral genomic replication and assembly. Moreover, compared with WT virus, the JEV prM-S78R-K79R-S81R mutant displayed attenuated virulence in a mouse model. Our findings may help to understand the mechanism of JEV replication, and would be useful in the development of novel JEV vaccines.

## RESULTS

### Mutations of S78R-K79R-S81R in prM increase prM/M ratio.

**FIG 1:**
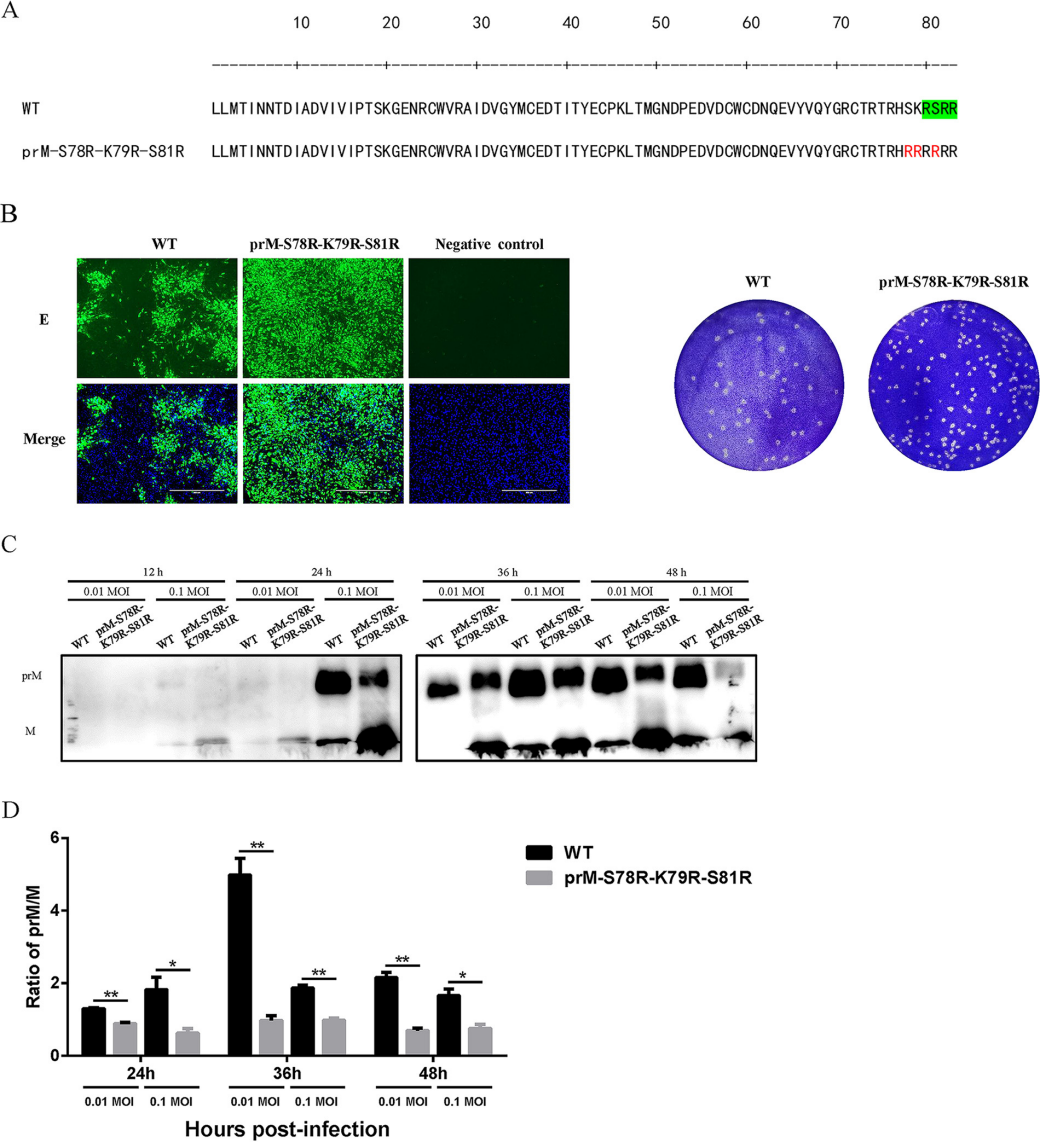
Mutations of S78R-K79R-S81R in furin cleavage sites and its nearby. (A) The furin cleavage sites in wild type prM (green in upper panel) and mutations of S78R-K79R-S81R in prM (red in lower panel). (B) IFA and plaque assay of WT and prM-S78R-K79R-S81R mutant virus. BHK-21 cells at 80% confluence were infected with WT and prM-S78R-K79R-S81R mutant virus, respectively. At 36-h postinfection, cells were fixed and subjected to IFA by using anti-E monoclonal antibody. Then cells were observed under inverted fluorescence microscope. Then the supernatants of transfected cell were subjected to plaque assay. (C, D) The prM or M protein expression of WT and mutant JEV in BHK-21 cells. BHK-21 cells were incubated with WT and mutant viruses at an MOI of 0.01 or 0.1. The cells were harvested at the indicated time points that were used to determine expression of viral proteins (C). The gray value of prM/M protein was analyzed by Image J (D). Asterisk (*) indicates a significant difference between WT and mutant virus (*, *P* < 0.05; **, *P* < 0.01).

Structural and functional mapping of the furin cleavage sites revealed the importance of furin and its preference (21, 23, 24). Previous studies in our laboratory have demonstrated that mutations in furin cleavage sites and nearby lead to alterations in ZIKV replication and virulence (22). Therefore, the similar mutant, JEV prM-S78R-K79R-S81R, was constructed using the full-length JEV cDNA clone. The furin cleavage sites in wild-type prM and mutations of S78R-K79R-S81R in prM have been shown in Fig. 1A. The BHK-21 cells were transfected with plasmid encoding full-length genome of WT or mutant JEV, and the results of indirect immunofluorescence (IFA) demonstrated the expression of prM-S78R-K79R-S81R mutant viral protein (Fig. 1B, left panel) and plaque assay with the supernatants of transfected cells demonstrating that the desired JEV prM-S78R-K79R-S81R mutant virus was rescued successfully (Fig. 1B, right panel). Furthermore, the cleavage of prM protein of JEV prM-S78R-K79R-S81R mutant was examined by Western blot assay. The results showed that the prM/M ratio of the prM-S78R-K79R-S81R mutant was obviously decreased than that of WT virus (Fig. 1C and D), indicating that the mutations of S78R-K79R-S81R in prM increase the cleavage efficiency of furin.

### Mutations of S78R-K79R-S81R in prM enhance JEV replication.

**FIG 2:**
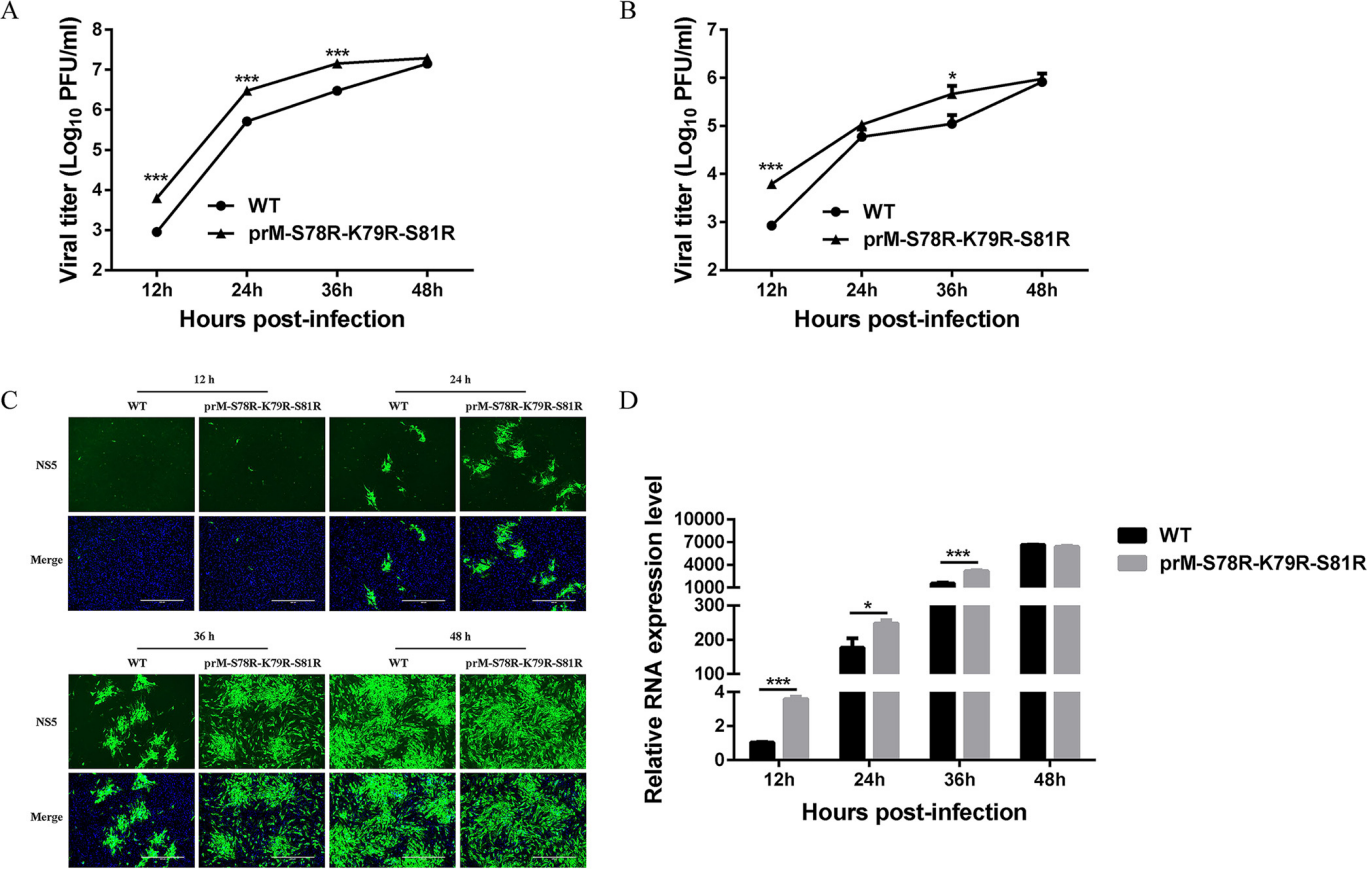
Characterization of JEV prM-S78R-K79R-S81R mutant. (A, B) Multistep growth curves of WT and mutant JEV in BHK-21 (A) and C6/36 cells (B). BHK-21 or C6/36 cells were incubated with WT and mutant at an MOI of 0.1. The cells and supernatants harvested at the indicated time points were used to determine the viral titers. The data were presented as means ± SD from three independent experiments. Asterisk (*) indicates a significant difference between WT and mutant virus (*, *P* < 0.05; ***, *P* < 0.001). (C, D) The levels of NS5 protein (C) and viral RNA (D) of JEV WT and mutant virus in BHK-21 cells. BHK-21 cells were incubated with WT and mutant virus at 0.1 MOI. At 12 hpi, 24 hpi, 36 hpi, and 48 hpi, the supernatants were removed and cells were subject to IFA and RT-qPCR assays. β-actin mRNA level was served as an internal control to determine the relative viral RNA expression level. Asterisk (*) indicates a significant difference between WT and mutant virus (*, *P* < 0.05; ***, *P* < 0.001).

In order to explore the role of prM mutation on JEV replication, BHK-21 and C6/36 cells were infected with WT and prM-S78R-K79R-S81R mutant virus at 0.1 multiplicity of infection (MOI), respectively. And the cells and supernatants were harvested at 12 h postinfection (hpi), 24 hpi, 36 hpi, and 48 hpi, and subjected to freeze thaw cycle, followed by determination of viral titers by plaque assay. The results showed that compared with WT virus, prM-S78R-K79R-S81R mutant generated more infectious progeny virus at different time points in both BHK-21 and C6/36 cells (Fig. 2A and B). Consistently, more cells expressing viral protein in prM-S78R-K79R-S81R mutant infected cells were observed than those in WT virus-infected cells (Fig. 2C), and more viral RNA were detected in prM-S78R-K79R-S81R mutant-infected cells at most time points than those in WT-infected cells (Fig. 2D), suggesting that the mutant replicates more efficiently than WT virus.

### Mutations of S78R-K79R-S81R in prM increase JEV propagation via promoting viral genomic replication and assembly.

**FIG 3:**
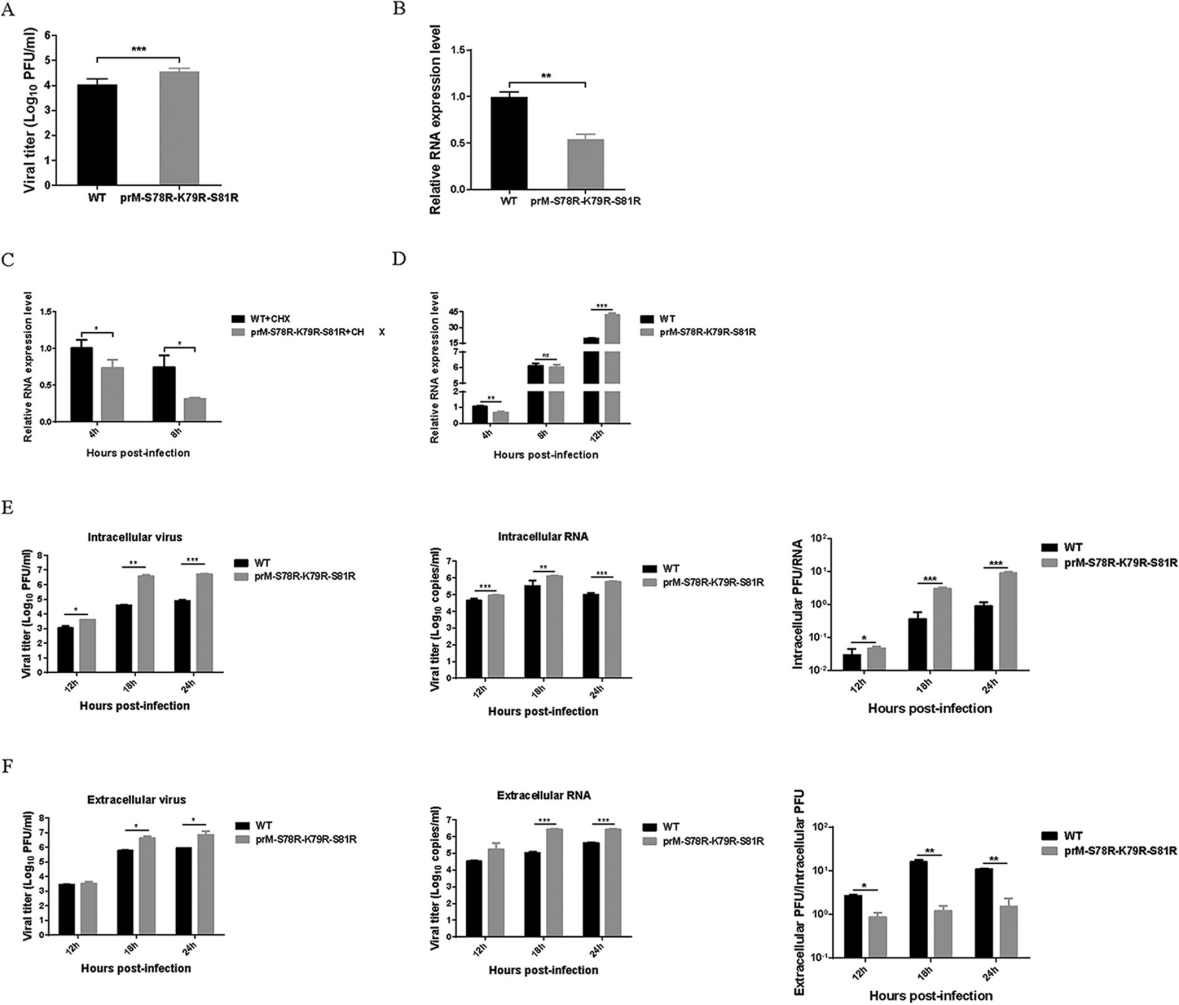
Mutations in furin cleavage sites of prM affect JEV genomic replication and assembly. (A) The attachment assay of WT and mutant JEV. BHK-21 cells were incubated with WT or mutant virus (5 MOI) at 4°C for 1 h. Then the cells were washed three times with PBS, and the viral titers attached to the cell surface (subject to freeze-thaw cycle) were measured by plaque assays. (B) The entry assay of WT and mutant JEV. BHK-21 cells were incubated with WT or mutant virus (5 MOI) at 4°C for 1 h and another 1 h at 37°C. Then the infected cells were stringently washed three times with PBS to remove free virus and another three times with an alkaline high-salt solution (1 M NaCl and 50 mM sodium bicarbonate [pH 9.5]) to remove surface-associated virus, and the level of viral RNA entering cell was measured by qRT-PCR. (C) The CHX assay of WT and mutant JEV. BHK-21 cells were incubated with 5 MOI of WT or prM-S78R-K79R-S81R virus at 37°C for 1 h, and the unattached viruses were removed at 1 hpi by washing three times with PBS. Then the cells were incubated with DMEM containing 2% FBS and CHX (100 μg/mL), and the cells were harvested at indicated time points. Finally, the intracellular viruses were quantified by measuring the viral RNA by qRT-PCR. (D) The replication assay of WT and mutant JEV. BHK-21 cells were infected with WT or mutant virus at (1 MOI) at 37°C for 1 h. Subsequently, the viral supernatants were removed, and cells were incubated with DMEM containing 2% FBS. Then the supernatants and cells were harvested at the indicated time points. Finally, the intracellular RNA was measured by qRT-PCR. (E, F) The assembly (E) and release assays (F) of WT and mutant JEV. BHK-21 cells were infected with WT or mutant virus at (1 MOI) at 37°C for 1 h. Subsequently, the viral supernatants were removed, and cells were incubated with DMEM containing 2% FBS. Then the supernatants and cells were harvested at the indicated time points. Intracellular and extracellular RNA were measured by qRT-PCR. Intracellular and extracellular viral titers were determined by plaque assay. Asterisk (*) indicates a significant difference between WT and mutant (*, *P* < 0.05; **, *P* < 0.01; ***, *P* < 0.001).

Next, the impact of mutations on viral life cycle was investigated. For analyzing viral attachment and entry, the BHK-21 cells were incubated with 5 MOI of WT or prM-S78R-K79R-S81R virus at 4°C for 1 h. After washing three times with phosphate buffered saline (PBS), the viruses attached to the cell surface were measured by plaque assay. The results revealed that more mutations attached to cells than WT virus (Fig. 3A), suggesting that the S78R-K79R-S81R mutations could increase the attachment of JEV to host cells.

Subsequently, cells were further incubated at 37°C for 1 h to initiate viral entry. Then the infected cells were stringently washed with PBS and an alkaline high-salt solution to remove free virus as well as cell surface-associated virus. Finally, the entry was evaluated by the level of intracellular viral RNA. However, the results reflected that more intracellular viral RNA were detected in WT-infected cells than those in mutant-infected cells (Fig. 3B), suggesting the mutations reduced the viral invasion. This result was further confirmed by treatment of the viral infected cells with the translation inhibitor, CHX, which also showed decreased viral RNA level in mutant-infected cells (Fig. 3C). Overall, the above results indicated that the viral attachment and entry are not the key steps that contribute to the increased replication of mutant virus.

To explore the effect of prM mutations on genomic replication of JEV, the RNA levels of JEV in WT or mutant virus-infected cells were measured at 4 hpi, 8 hpi, and 12 hpi. It was found that the RNA levels of mutant virus at 4 hpi were significantly lower than those of WT virus; however, there is no significant difference of viral RNA levels at 8 hpi, and a much higher RNA level of mutant virus than that of WT virus was detected at 12 hpi (Fig. 3D). These results indicates that the mutations on prM protein may promote the replication efficiency of JEV genomic RNA.

To further analyze the impact of mutations on viral assembly and release, BHK-21 cells were incubated with 1 MOI of WT or prM-S78R-K79R-S81R virus at 37°C for 1 h, and the unattached viruses were removed at 1 hpi by washing three times with PBS. Followingly, the supernatants and cells were harvested at different time points. To measure the efficiency of intracellular viral assembly, the intracellular viral PFU/RNA ratios were measured, and the results showed that the intracellular PFU/RNA ratio derived from mutant were 2-, 8-, and 10-fold higher than WT virus at 12 hpi, 18 hpi, and 24 hpi, respectively (Fig. 3E), suggesting that the assembly of mutant was more efficient than that of WT virus. Meanwhile, the extracellular PFU/intracellular PFU ratios were measured to estimate viral release. Interestingly, the results revealed that the extracellular PFU/intracellular PFU ratios derived from mutant were slightly lower than WT virus, suggesting that the mutant virus was less efficient in viral release (Fig. 3F). Taken together, these results indicate that the mutations may increase the propagation of JEV via promoting both the viral genomic replication and assembly.

### Mutations of S78R-K79R-S81R in prM affect plaque morphology of JEV.

**FIG 4:**
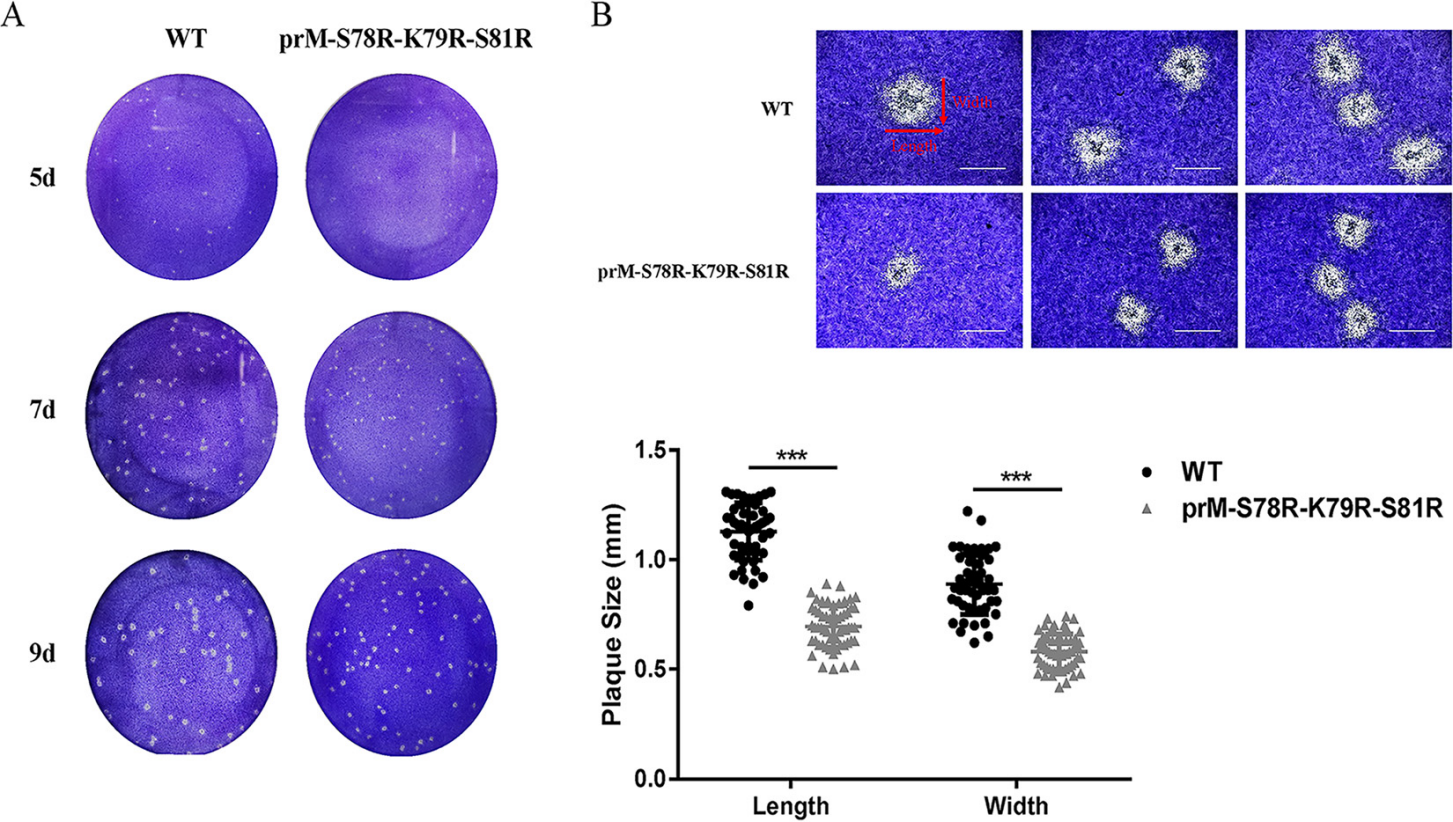
Plaque morphology of WT and mutant JEV in BHK-21 cells. (A) Plaque morphology of WT and mutant JEV in BHK-21 cells. BHK-21 monolayers were infected with WT or mutant virus, and then stained with crystal violet at 5 dpi, 7 dpi, and 9 dpi. (B) The plaques of WT and mutant virus in BHK-21 cells at 9 dpi. The plaques with clearer morphology (9 dpi) were randomly selected by microscope (upper panel). The plaque size (*n* ≥ 50) was analyzed by GraphPad Prism 7 (lower panel). The length and width of plaques were labeled by arrows. Asterisk (*) indicates a significant difference between WT and mutant virus (***, *P* < 0.001).

In order to explore the difference of plaque morphology between WT and prM-S78R-K79R-S81R mutant virus, BHK-21 cells were infected with the individual viruses and stained on 5 days postinfection (dpi), 7 dpi, and 9 dpi, respectively. Interestingly, smaller size of plaques formed by prM-S78R-K79R-S81R mutant was observed, compared with those formed by WT virus (Fig. 4A). Length and width of plaques with clearer morphology was measured by using Image J software. The results revealed that the average diameter of plaques formed by prM-S78R-K79R-S81R mutant were about 78% of the WT in length and 82% in width (Fig. 4B), indicating the mutations may attenuate the cytopathogenicity of JEV.

### Mutations of S78R-K79R-S81R in prM attenuate the virulence of JEV without affecting viral neuroinvasiveness.

**FIG 5:**
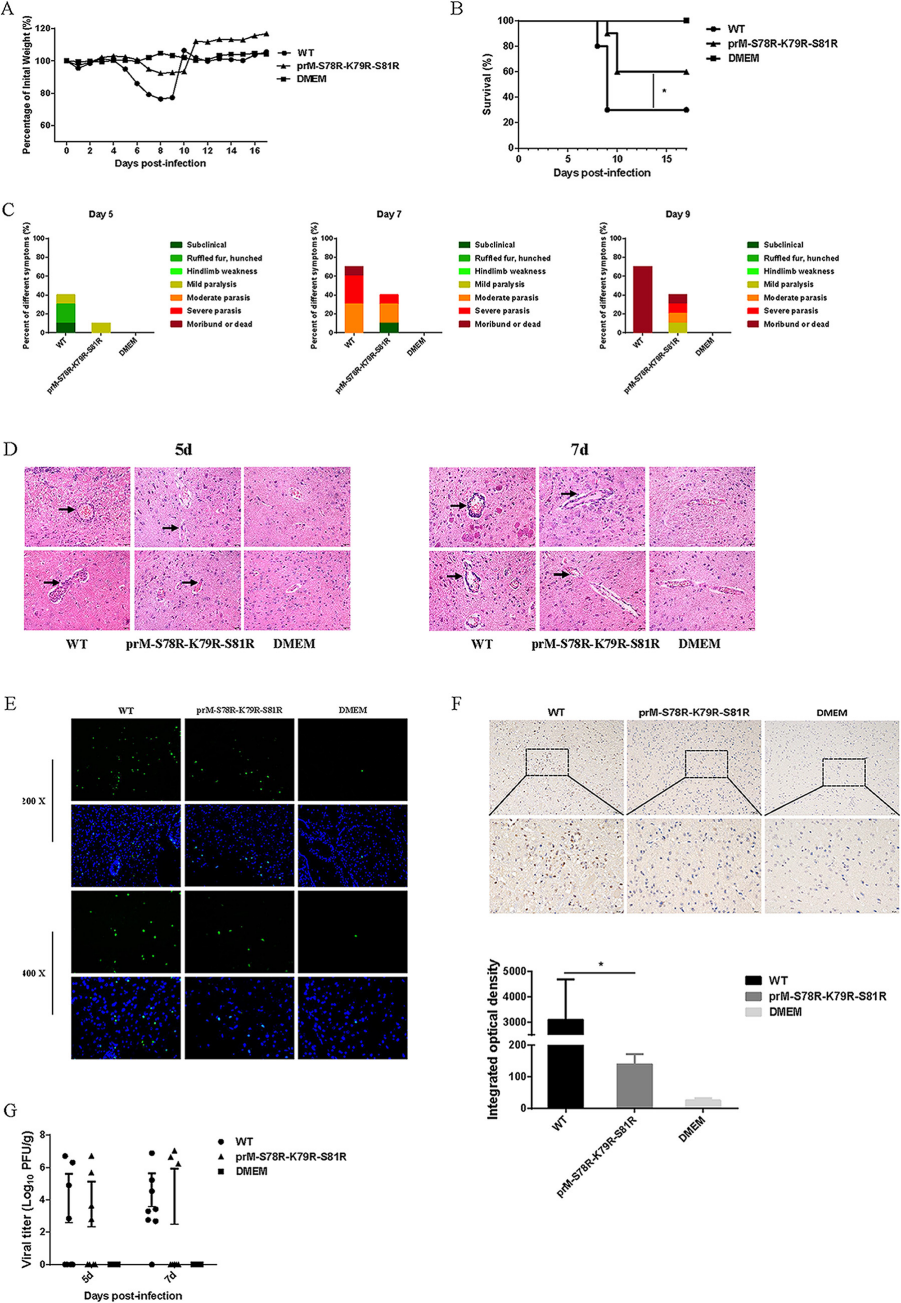
Neuroinvasiveness of JEV prM-S78R-K79R-S81R mutant. Five-week-old C57/BL6 mice (*n* = 10 per group) were inoculated with 10^6^ PFU WT and mutant virus via intraperitoneal injection, respectively. And the control group was injected with isopycnic DMEM. (A to C) Body weight (A), survival curve (B), and clinical symptoms (C) were monitored. (D) The pathological changes in the brain tissues were examined by H&E staining, represented by black arrows. (E) The apoptotic neurons in the mouse brain on 7 dpi were detected using the TUNEL assay kit. (F) Immunohistochemistry (IHC) analysis of brain was performed to determine the expression of cleaved caspase 3 protein (upper panel). Integrated option density (IOD) analysis was performed to quantify the results of staining (lower panel). Asterisk (*) indicates a significant difference between WT and mutant (*, *P* < 0.05). (G) Viral titers in the brain tissues at 5 dpi and 7 dpi were determined by plaque assay. Each data point represents one mouse.

To clarify the effect of the mutations in prM on JEV virulence, two groups of 10 C57BL/6 mice were intraperitoneal (i.p.) injected with 10^6^ PFU WT and mutant virus, respectively, and the control group was injected with isopycnic Dulbecco’s modified Eagle’s medium (DMEM). Mouse survival rate and clinical symptom were monitored daily for 3 weeks. The average weight of mice in the WT and mutant group gradually decreased from the 5th day, and the average weight reached the lowest on the 8th day (Fig. 5A). The mice in the WT-infected group died the 8th day, and the final survival rate was 30%. Meanwhile, the mice in the mutant-infected group died the 9th day, and the final survival rate was 60% (Fig. 5B). In order to present clinical symptoms, the behavioral signs of mice in all the experimental groups were evaluated. The results showed that compared with the WT-infected group, the mutant-infected group had milder symptoms on 5 dpi, 7 dpi, and 9 dpi (Fig. 5C). These results suggest the attenuated virulence of mutant JEV, compared with that of WT virus.

To assess the pathology of the brain tissue, hematoxylin-eosin (H&E) staining, immunofluorescence (IF), and immunohistochemistry (IHC) were conducted on 5-dpi or 7-dpi collected brain samples. The histopathological changes of the cerebrum in the WT-infected group indicated perivascular cuffing and meningitis, but these indicators of encephalitis were reduced in the mutant-infected group (Fig. 5D). In order to explore the difference in neuron damage between the WT-infected and mutant-infected group, brain samples processed on day 7 postinfection were subjected to a TUNEL assay. The results revealed that the numbers of TUNEL-positive cells in the WT-infected mouse brain were higher than those in the mutant-infected mouse brain (Fig. 5E). Furthermore, the activated caspase 3 in brain tissue collected on day-7 postinfection were detected by IHC assay, and the results showed that the level of cleaved caspase3 protein in brain tissue of mutant-infected mice were significantly lower than that in brain of WT-infected mice (Fig. 5F), suggesting alleviated neuron death in mutant-infected mice than that in WT-infected mice.

To investigate whether the reduced lethality and neuronal damage of mutant-virus-infected mice was caused by attenuated neuroinvasiveness of JEV, viral loads in the brain tissues were measured by plaque assay. However, similar viral titers were detected in brain tissues of WT- and mutant-virus-infected mice on 5 dpi or 7 dpi (Fig. 5G). This result demonstrated that the mutations in prM did not affect the neuroinvasiveness of JEV, indicating the reduced lethality and neuronal damage of mutant-virus-infected mice may be a result of the attenuated neurovirulence of mutant JEV.

### Mutations of S78R-K79R-S81R in prM attenuate the neurovirulence of JEV.

**FIG 6:**
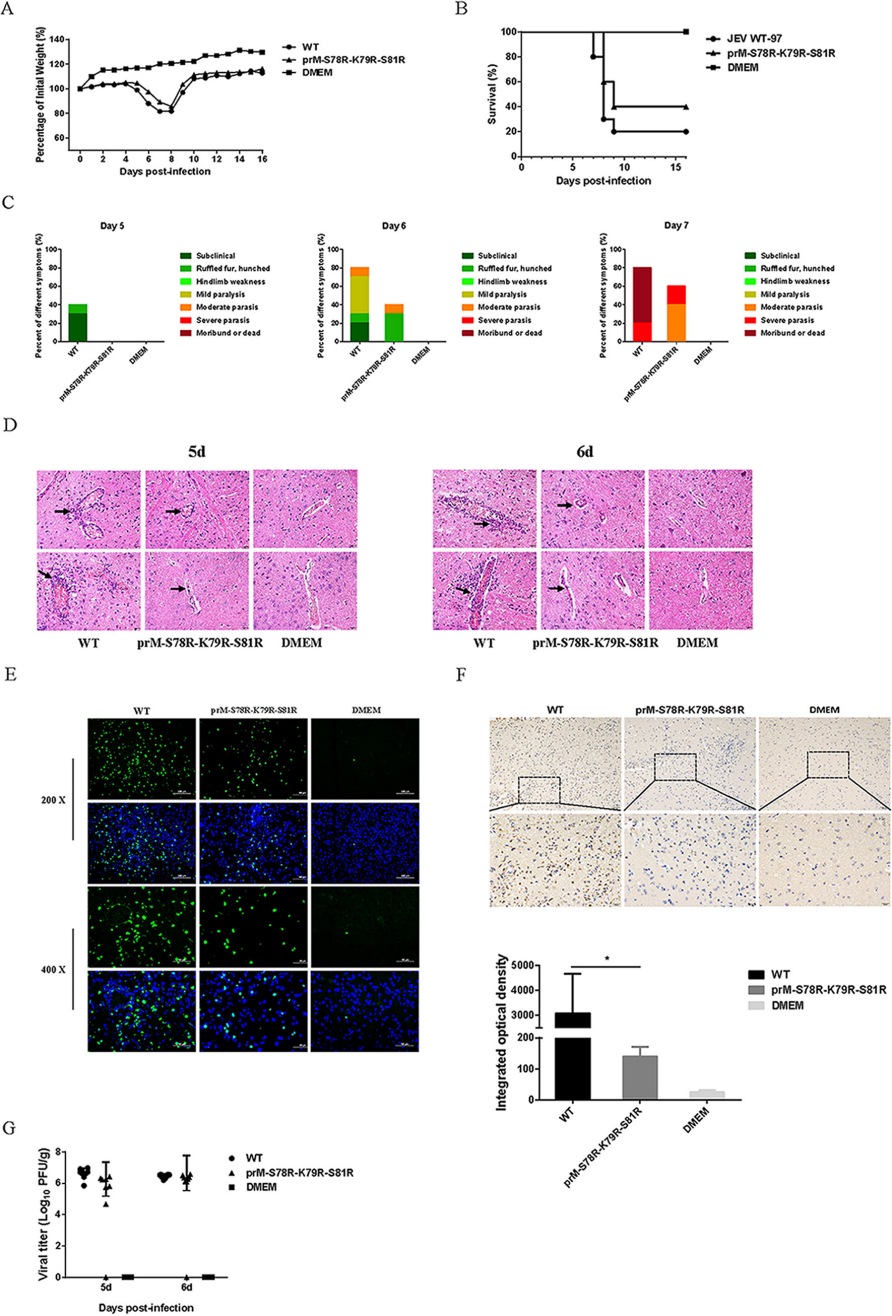
Neurovirulence of JEV prM-S78R-K79R-S81R mutant. Five-week-old C57/BL6 mice (*n* = 10 per group) were inoculated with 10^3^ PFU WT and mutant viruses via intracerebral injection. And the control group was injected with isopycnic DMEM. (A to C) Body weight (A), survival curve (B), and clinical symptoms (C) were monitored. (D) The pathological changes in the brain tissues were examined by H&E staining, represented by black arrows. (E) The apoptotic neurons in the mouse brain on 6 dpi were detected using the TUNEL assay kit. (F) Immunohistochemistry (IHC) analysis of brain to determine the expression of cleaved caspase3 protein (upper panel). Integrated option density (IOD) analysis was performed to quantify the results of staining (lower panel). Asterisk (*) indicates a significant difference between WT and mutant virus (*, *P* < 0.05). (G) Viral titers in the brain tissues at 5 dpi and 6 dpi were determined by plaque assay. Each data point represents one mouse.

To evaluate the neurovirulence of the mutant and WT virus, two groups of 10 C57BL/6 mice were intracranial injection (i.c.) inoculated with 10^3^ PFU WT or mutant virus, respectively. The global trend of weight change in the intracerebral injection groups was similar to that of the intraperitoneal injection groups (Fig. 6A). And compared with WT-infected mice, the mutant-infected mice showed a higher survival rate (Fig. 6B) and milder symptoms (Fig. 6C). In addition, brain samples on day-6 postinfection in WT-infected and mutant-infected groups were subjected to HE staining, TUNEL, and IHC assay. The results showed that the histopathological changes of the cerebrum in the mutant-infected group were more alleviative than those in the WT-infected group (Fig. 6D), and the numbers of TUNEL-positive cells and the level of activated caspase 3 protein in the brain of mutant-infected mice were reduced compared with those in the brain of WT-infected mice (Fig. 6E and F). Furthermore, the viral loads in the brain of mutant-infected mice were similar to that in WT-infected mice (Fig. 6G). These results indicated the attenuated neurovirulence of mutant virus.

## DISCUSSION

As an important vector-borne zoonotic disease, Japanese encephalitis virus causes a serious threat to the people’s health worldwide. Many studies have demonstrated that the prM/M and E proteins play important roles in the entry, assembly, and secretion of flavivirus (25–28). Presently, there are many studies on E protein, and its function and mechanism are relatively clear (29–35). However, the studies on the cleavage of prM to M and the function of prM/M are still limited. The previous studies have demonstrated that the R-X-R/K-R motif alone is insufficient for predicting furin proteolysis of the substrate, and it also reveals the importance of both short-range (P4 to P1) and long-range (P7 to P6) interactions in defining furin cleavage specificity (21, 23, 24). Therefore, the JEV prM-S78R-K79R-S81R mutant was constructed to investigate the relationship between furin and viral replication or pathogenicity based on the preference for furin cleavage (21) and the previous study about prM cleavage of ZIKV in our laboratory (22).

Our study showed that compared with WT JEV, the prM-S78R-K79R-S81R mutant generated significantly more viral RNA and infectious virions (Fig. 2), indicating that prM cleavage is closely related to viral replication. In previous studies, the relationship between furin and viral replication and its importance has been explored. For example, the entecavir combined with furin inhibitor simultaneously reduces hepatitis B virus replication and E antigen secretion (36); the acquisition of furin cleavability by avian paramyxovirus serotype 7 (APMV-7) results in syncytium formation and increased virus yield *in vitro* but does not alter virus yield, tropism, or virulence in chickens (37). Also, the SARS-CoV-2 replication is strongly inhibited by the synthetic furin inhibitor MI-1851 in human airway cells (38). Interestingly, in our study, more significant replication difference between prM-S78R-K79R-S81R mutant and WT was observed in BHK-21 cells than that in C6/36 cells (Fig. 2A and B), indicating a species selectivity of its role in viral replication. However, the mechanism is still needed for further study.

Subsequently, the effect of mutations in furin cleavage sites on different steps of viral life cycle were examined. The results showed that the mutant displayed increased attachment ability than the WT virus (Fig. 3A), in accordance with a previous study about the Dengue virus (39). But compared with WT virus, the mutant virus was less efficient in viral entry (Fig. 3B and C), and the mechanism of which is still needed for further study. Our study also revealed that the mutant displayed an enhanced ability of assembly than the WT virus (Fig. 3E), which is also in consistence with the previous study (40). However, compared with the WT virus, the release efficiency of the mutant virus was reduced (Fig. 3F), and its possible mechanism is also needed for further exploration.

It has been known that the viral plaque morphology is correlated with not only viral spread but also its cytopathogenicity (35, 41–43). Our study revealed that the prM-S78R-K79R-S81R mutant virus increased viral yield but displayed smaller plaques than the WT virus (Fig. 4), indicating that the mutant virus may possess lower cytopathogenicity and attenuated virulence. The enhanced replication and spread of the mutant virus (Fig. 2) may be due to reduced cell damage (in line with smaller plaque) in the mutant virus, which creates an environment more suitable for viral replication and spread. It is also a strategy for accelerating transmission and escaping host immunity during viral evolution.

To further explore the difference of virulence between the WT and mutant virus, the *in vivo* experiments with mice injected in intraperitoneal and intracerebral with WT or mutant virus were conducted. In both the intraperitoneal and intracerebral injection experiments, the mutant-infected group showed a significantly higher survival rate, a coincidence with the results of histopathological changes of the cerebrum and neuronal damage of mice (Fig. 5 and 6). These results are also consistent with the previous studies about the prM protein in ZIKV (22, 44). Generally, the above results indicated that the mutations in prM protein attenuate the virulence of JEV.

In summary, the increased cleavage of prM protein promotes propagation of JEV via increasing the viral genomic replication and assembly but attenuates its virulence *in vivo*. These findings would not only help to understand the function of prM/M protein and pathogenesis of JEV, but also provide new clues for vaccine development of flavivirus.

## MATERIALS AND METHODS

### Cells, viruses, and antibodies.

BHK-21 (Baby hamster kidney) cells were cultured in DMEM (Sigma-Aldrich, USA), supplemented with 10% fetal bovine serum (FBS, Every Green, China), 100 IU/mL penicillin and 100 μg/mL streptomycin at 37°C with 5% CO2. Aedes albopictus C6/36 cells were cultured in DMEM (Biological Industries, USA), supplemented with 10% FBS (Biological Industries, USA), 100 IU/mL penicillin, and 100 μg/mL streptomycin at 28°C with 5% CO2. The wild-type JEV (GenBank no. AB196925) and prM-S78R-K79R-S81R mutant virus were rescued and stored in our laboratory. Monoclonal mouse anti-JEV M MAb, E Mab, and NS5 MAb were prepared and preserved in our laboratory.

### Plasmid construction and viral rescue.

The infectious clone of WT JEV was preserved in our laboratory. Based on the WT plasmid, the prM-S78R-K79R-S81R mutant plasmid was constructed using PCR-based site-directed mutagenesis. Briefly, the PCR products were amplified by using different primers (F1/R1, F2/R2) with the template of WT plasmid, and the varied PCR products were fused by fusion PCR. Then the WT plasmid and mutant DNA fragments were digested with the same restriction enzyme of *Apa* I and *Sac* I. Finally, the WT vector and the mutant DNA fragments were ligated by T4 DNA ligase. Finally, the desired mutant plasmid was constructed. All plasmids were subject to nucleotide sequencing and all primers were listed in Table 1.

**TABLE 1:** Primers used in this study

Primers	Sequences (5′–3′)	Application
F1	ACCAGGAGGGCCCGGTAAAAAC	Mutant clone
R1	ACGGATCTCCTCCTTCGCCTCCTATGCCTGGTC	
F2	GACCAGGCATAGGAGGCGAAGGAGGAGATCCGT	
R2	CTGCCAGTCTCTGAGCTCCCTT	
JEV-C-F	GGCTTTTATCACGTTCTTCAAGTTT	Relative qPCR
JEV-C-R	TGCTTTCCATCGGCCTAAAA	
β-actin-F	CACTGCCGCATCCTCTTCCTCCC	
β-actin-R	CAATAGTGATGACCTGGCCGT	
JEV-E-F	TGGTTTCATGACCTCGCTCTC	Absolute qPCR
JEV-E-R	CCATGAGGAGTTCTCTGTTTCT	
Probe	CCTGGACGCCCCCTTCGAGCACAGCGT	

For viral rescue, BHK-21 cells seeded in 6-well plates were transfected with the WT and mutant plasmids using NeofectTM DNA transfection reagent (Neofect biotech, China) according to the manufacturer’s instruction. The rescued viruses were individually designated as WT and prM-S78R-K79R-S81R. The supernatants of transfected cells were harvested at 96-h posttransfection, designated as passage 1 (P1) of the rescued viruses. The P1 was continuously passaged to the third generation (P3), followed by viral titration on BHK-21 cells, and the viruses were stored at −80°C for later use. All viruses (P3) were subject to nucleotide sequencing.

### Viral plaque assay and growth kinetics.

The WT and mutant viruses were serially 10-fold diluted in DMEM, and the diluted viruses were incubated with BHK-21 cells in 24-well plates. After 1 h at 37°C, the supernatants were removed, and the cells were washed three times with serum-free DMEM and overlaid with 1 mL DMEM containing 2% sodium carboxymethyl cellulose and 2% FBS. After incubation for 5 days at 37°C, the cells were fixed with 10% formaldehyde and stained with 0.6% crystal violet solution. Eventually, the visible plaques were counted, and the viral titers (PFU/mL) were calculated.

To determine the growth properties of WT and mutant viruses, the BHK-21 or C6/36 cells in 6-well plates were infected with virus at a MOI of 0.1. After 1 h at 37°C or 28°C, the supernatants were removed, and the cells were washed three times with serum-free DMEM and then incubated with 2 mL DMEM containing 2% FBS. The cells and supernatants at 12 hpi, 24 hpi, 36 hpi, and 48 hpi were harvested and titrated. Each time point was independently repeated three times.

### Indirect immunofluorescence.

BHK-21 cells seeded in 24-well plates were infected with WT and mutant viruses. After 1 h at 37°C, the supernatants were removed, and the cells were washed three times with serum-free DMEM, followed by incubation with 500 μL DMEM containing 2% FBS at 37°C for 36 h. The cells were washed once with PBS, and fixed with cold methanol for 10 min. After washing with PBS, cells were then permeabilized with 0.1% Triton X-100 in PBS at room temperature for 10 min. After blocking with 1% bovine serum albumin (BSA) in PBS for 30 min, the cells were incubated with anti-JEV E or NS5 protein monoclonal antibody for 1 h. After washing three times with PBS, cells were incubated with an Alexa Fluor 488-conjugated secondary antibody (Invitrogen, USA) for 30 min. Cell nuclei were stained with 4’, 6-diamidino-2-phenylindole (DAPI, Invitrogen, USA) for 10 min. Staining was observed using a fluorescence microscope (Zeiss, Germany).

### Western blotting.

Cells were harvested and incubated in lysis buffer (Beyotime Biotechnology, Shanghai, China) containing protease inhibitor for 30 min on ice, and the supernatants were collected after centrifuging at 12,000 rpm for 10 min at 4°C. Protein concentrations were measured with a bicinchoninic acid protein assay kit (BCA, Thermo Scientific, USA). The proteins were separated by SDS-PAGE, transferred to nitrocellulose membrane, incubated with primary and secondary antibodies, and visualized with an enhanced chemiluminescence system (Tanon, China).

### RNA extraction and real-time reverse transcription (RT)-PCR.

Total RNA was extracted from cells using TRIzol reagent (Invitrogen, USA), and the first-strand cDNA was synthesized using the ABScript II cDNA First-Strand Synthesis Kit (ABclonal, China). Relative quantitative PCR (qPCR) was performed using 2X University SYBR green Fast qPCR Mix (ABclonal, China). Primers JEV-C-F/R were used for relative analysis of viral replication. Primers β-actin-F/R were used for quantification of β-actin mRNA that served as an internal control. The absolute qPCR was performed with the primers JEV-E-F/R and probe. The standard curve was performed with 10-fold serial diluted plasmid containing JEV E gene. All the primers were listed in Table 1. The qPCR was performed with the QuantStudio 6 Flex PCR System (Applied Biosystems, USA).

### Quantification of intracellular and extracellular infectious virions.

For analyzing viral attachment, the BHK-21 cells were incubated with 5 MOI of WT or prM-S78R-K79R-S81R virus at 4°C for 1 h, allowing the viruses to attach to the cell surface without entering. Then the cells were washed three times with PBS to remove unattached virus, and the viruses attached to the cell surface were released by freeze-thaw cycle. The viral titers attached to the cell surface were measured by plaque assay.

For viral entry assay, the BHK-21 cells were incubated with 5 MOI of WT or prM-S78R-K79R-S81R virus at 4°C for 1 h. Then the cells were further incubated at 37°C for 1 h to initiate viral entry. Subsequently, the infected cells were stringently washed three times with PBS to remove free virus and washed for another three times with an alkaline high-salt solution (1 M NaCl and 50 mM sodium bicarbonate [pH 9.5]) to remove surface-associated virus. Finally, the internalized viruses were quantified by measuring the viral RNA by qRT-PCR. Moreover, in order to further exclude the effect of replication on viral entry, the cycloheximide (CHX) experiments were performed. Briefly, the BHK-21 cells were incubated with 5 MOI of WT or prM-S78R-K79R-S81R virus at 37°C for 1 h, and the unattached viruses were removed at 1 hpi by washing three times with PBS. Then the cells were incubated with DMEM containing 2% FBS and CHX (100 μg/mL), and the cells were harvested at indicated time points. Finally, the intracellular viruses were quantified by measuring the viral RNA by qRT-PCR.

For viral assembly and release assays, the BHK-21 cells were incubated with 1 MOI of WT or prM-S78R-K79R-S81R virus at 37°C for 1 h, and the unattached viruses were removed at 1 hpi by washing three times with PBS. The supernatants or cells were harvested at different time points for plaque or qRT-PCR assay to determine the intracellular/extracellular infectivity.

### Mouse experiments.

All mouse experiments were performed according to protocols approved by the Animal Care and Ethics Committee of Huazhong Agricultural University, under the number HZAUMO-2021-0163. All protocols adhered to the Guide for the Care and Use of Laboratory Animals. Four-week-old C57BL/6 mice were purchased from Animal Center of Huazhong Agricultural University, housed in an environmentally controlled room, and maintained on standard laboratory food and water throughout the study. To assess the neuroinvasiveness of the WT and mutant viruses, 10 mice per group were intraperitoneally (intraperitoneal injection, i.p.) inoculated with 100 μL diluent virus (10^6^ PFU). Mouse survival and clinical symptom were monitored daily for 3 weeks. At 5 dpi and 7 dpi, mice from each group were sacrificed, and the brains were used for determination of viral titers. Ketamine-xylazine (0.1 mL per 10 g of body weight) was used to anesthesia the treated mice. Brain tissues were collected then embedded in paraffin for coronal sections. The sections were used for H&E staining, immunofluorescence IF, and immunohistochemistry IHC.

To evaluate the neurovirulence of WT and mutant viruses, 10 mice per group were intracerebrally (intracranial injection, i.c.) inoculated with 20 μL diluent virus (10^3^ PFU). Mouse survival and clinical symptom were monitored daily for 3 weeks. At 5 dpi and 6 dpi, mice from each group were sacrificed, and the brains were used for determination of viral titers. Ketamine-xylazine (0.1 mL per 10 g of body weight) was used as anesthesia for the treated mice. Brain tissues were collected then embedded in paraffin for coronal sections. The sections were used for H&E staining, IF, and IHC.

### Statistical analysis.

All experiments were performed at least three independent replicates. All data were analyzed by GraphPad Prism 7. The measured values were expressed as the mean with standard deviation (SD). Differences were analyzed for statistical significance using two-tailed *unpaired t test* for two groups. Differences were considered statistically significant at a value of *P* < 0.05.

### Ethical statement.

The animal study was reviewed and approved by The Scientific Ethic Committee of Huazhong Agriculture University (HZAUMO-2021-0163). Written informed consent was obtained from the owners for the participation of their animals in this study.
